# Influence of Heat Stress on Milk Production, Milk Quality, and Somatic Cell Count in Galicia (NW Spain)

**DOI:** 10.3390/ani15070945

**Published:** 2025-03-25

**Authors:** Roberto Besteiro, Ramiro Fouz, Francisco Javier Diéguez

**Affiliations:** 1 Centro de Investigacións Agrarias de Mabegondo, Axencia Galega de Calidade Alimentaria Xunta de Galicia, 15318 A Coruña, Spain; 2 Área de Produción Animal, Facultade de Veterinaria, Universidade de Santiago de Compostela, 27002 Lugo, Spain; ramiro.fouz@usc.es (R.F.); franciscojavier.dieguez@usc.es (F.J.D.)

**Keywords:** climate change, dairy industry, production traits

## Abstract

This study analyses the effect of heat stress (HS) on milk production, composition, and somatic cell count in Holstein cows in Galicia. For this purpose, the study uses the temperature and humidity index (THI) obtained from the official network of weather stations and data from the official milk control between 2016 and 2021. For milk production, critical THI thresholds were established at THI = 72, while the somatic cell count increased when THI exceeded 78. For the fat and protein components of milk, the critical thresholds are lower, at 64 and 63, respectively. The analysis shows that for each unit increase in THI above the critical threshold, up to 0.249 kg of milk, 0.008 kg of protein, and 0.006 kg of fat can be lost per cow per day. HS has a greater impact on milk composition than on volume, particularly on protein content. In addition, it was observed that the impact of HS on milk production can last between 7 and 12 days. Similarly, consecutive cumulative days of HS significantly impact both milk production and quality, especially in higher productivity stages multiparous cows. Although Galicia has a less extreme climate than other regions, the presence of moderate HS during the warmer months causes significant economic losses in the dairy sector.

## Simple Summary

This study investigates how heat stress (HS) affects milk production, composition, and somatic cell count in Holstein cows in Galicia. The researchers used the temperature-humidity index (THI) to assess the climate conditions over several years (2016–2021) and their impact on dairy farming. The study found that when the THI exceeded certain thresholds, milk production decreased, and the quality of milk—especially its protein and fat content—was significantly affected. For example, for every unit increase in THI above the critical level, cows can lose up to 0.249 kg of milk per day. This shows that heat stress has a more pronounced effect on the composition of the milk than its volume. The impact of heat stress can last between 7 and 12 days and become more harmful if heat stress is accumulated over consecutive days, particularly for cows in the middle of their lactation cycle. While Galicia’s climate is milder than other regions, even moderate heat stress during the warmer months leads to substantial economic losses in the dairy sector. These findings emphasise growing concerns over climate change, as rising temperatures could further exacerbate the challenges faced by the dairy industry, highlighting the need for strategies to protect livestock and ensure sustainable milk production.

## 1. Introduction

The increasing milk yields achieved in cattle, together with the parallel increase in their intake capacity, cause them to generate greater metabolic heat, produced during ruminal fermentation, which can hinder their ability to dissipate heat under heat stress conditions. Heat stress (HS) is the animal’s physiological response to the environment when it produces more heat than it can dissipate. In humid environments, as temperatures rise, the ability of livestock to dissipate heat through evaporation is considerably more impaired than in areas with lower humidity. Humidity is therefore the limiting factor for HS in humid climates, whereas temperature tends to be the limiting factor in dry climates [1].

Environmental conditions are a major contributor to HS and are relatively easy to measure and monitor compared to measurable parameters in the animal itself. For this reason, HS indicators based on environmental parameters are used to assess their effect on animal welfare and health, as well as to relate this to any impact on production. The temperature and humidity index (THI) combines the effects of ambient temperature and relative humidity to which the animal is exposed [2], combining the impact of these two factors associated with HS into a single value.

Physiological changes linked to heat stress are often accompanied by behavioural alterations in the animal, such as reduced activity to conserve energy and minimise heat production [3], reduced time lying down to increase the body surface area available for heat loss, reduced rumination time, and increased respiratory rate [4]. In addition, livestock reduce their feed intake to reduce metabolic heat production [5]. These behavioural changes increase the risk of ruminal acidosis and lameness [3], thereby affecting milk production [1,6].

As production conditions, as well as temperature and humidity, vary between geographical regions, studies are needed to quantify the effect of heat stress on milk production, providing local benchmarks in terms of animal welfare and farm productivity, especially in a context where climate change is intensifying hot spells. This type of study, such as this one conducted in the north-western region of the Iberian Peninsula, provides data for one of the most important dairy-producing regions in Europe [7].

Operating with high-yield cows, averaging 10,000 kg per lactation at 305 days and an annual increase in average yield per cow of approximately 1.9% [8], climate change conditions with increased temperatures and unusual weather phenomena make HS a growing concern in regions such as Galicia. This region is experiencing an increase in the frequency, duration, and intensity of hot spells [9], a scenario that justifies the importance of studying and addressing the impact of climate change in this area.

In summary, we hypothesize that there are critical THI thresholds beyond which production and quality parameters deteriorate, and that the accumulation of consecutive heat stress days exacerbates these negative effects. Furthermore, cows in mid-lactation and those with higher milk yields will be more susceptible to production and compositional losses under heat stress compared to cows in early or late lactation. This study seeks to quantify these thresholds and assess the persistence of heat stress effects over time, contributing to region-specific guidelines for heat stress mitigation in temperate climates.

The specific aim of the study was, therefore, to analyse the effect of HS (measured as THI) on the production, composition, and somatic cell count of milk from Holstein cows in Galicia, Spain.

## 2. Materials and Methods

### 2.1. Population, Study Animals, and Climate Data

The dataset analysed corresponds to data from the Official Dairy Yield Control developed by the Galician Dairy Control Centre (Centro Gallego de Control Lechero; CEGACOL v.01.00.07) between January 2016 and December 2021 in the autonomous community of Galicia (northwestern Spain). The initial database contains the records of daily tests (TD) conducted on cows housed on farms with between 65 and 76 animals on average (the typical size in the region), fed mainly with fodder and feed, and with various milking systems (twice daily, three times daily, or a robotic milking system). Every month, the values in kg of the daily milk yield (MY), the percentage of fat and protein, and the somatic cell count (SCC, cells/mL × 1000) expressed through a linear score (LS) [log2 (SCC/100) + 3] were collected. Then, the daily yields in kg of fat and protein were obtained, as was the energy-corrected milk (ECM) production value according to the International Farm Comparison Network equation [10], based on a fat value of 4% and a protein figure of 3.3%.

Climate information was obtained from the MeteoGalicia weather station network, belonging to the regional government (Xunta de Galicia), for the same period of time. Subsequently, using the National Research Council (1971) equation, the maximum daily temperature-humidity index (THI) was calculated for the 46 weather stations closest to the farms in the production records database [11].

*THI* = (1.8 × *maxT* + 32) − (0.55 − 0.0055 × *minH*) × [(1.8 × *maxT* + 32) − 26] (1)

where THI is the value of the temperature-humidity index, maxT is the maximum daily ambient temperature at 1.5 m altitude, and minH is the minimum daily relative humidity at 1.5 m altitude. The maximum THI variant was used, as it correlates better with production losses [12], and is a better estimate of the real average THI of the herds [13]. It was decided to omit the THI value on the day of the milk testing, as many of these monitoring events take place in the morning, when the maximum temperatures of the day have not yet been reached. In addition, THI values were obtained from the day before to 12 days prior to the test day (TD) in order to describe how the impact of HS evolves over time. This 12-day window was chosen based on data published in the literature [14,15].

Those records with THI values >83 were later eliminated due to the low quantity of data. Also, only records from Holstein cows between 4 and 305 days in milk (DIM), with a lactation number (LN) between the first and eighth, and with at least two TD per lactation and two lactations were selected. Finally, outliers in each LN factor group were removed using the interquartile range method (three times the IQR). After this cleaning process, a database with 457,165 records for 21,779 cows from 175 farms was obtained. Due to the work-life balance of the dairy control staff, production records are only available for 11 months of the year, with information from the month of August only in 2020.

The THI values were categorised into 50 classes, corresponding to one class for each THI unit from THI = 33 to THI = 83. The LN was divided into 6 classes (1: first lactation, 2: second lactation, 3: third lactation, 4–5: fourth or fifth lactation, 6–8: sixth to eighth lactation). Similarly, the variable relating to the milking class (MC) was categorised into the following groups: two milkings per day and three milkings per day or a robotic milking system.

### 2.2. Statistical Analysis

Two different methods were used to study the effect of HS on milk production and composition. Firstly, the threshold THI level at which production losses occurred was determined and these losses were characterised. Secondly, the influence successive HS days had on the variables studied was analysed.

To determine the THI thresholds for MY, protein and fat percentage, protein and fat yield, LS, and ECM, the following linear mixed model was fitted:

*Yijklmn* = *THIi* + *LNj* + *DIMQ* + *LNj* × *DIMQ* + *MCk* + *yml* + *herd*(*cow*(*LN*))*m* + *eijklmn* (2)

where Yijlkmn is the value of the production parameter to be analysed (MY, protein and fat percentage, protein and fat yield, LS or ECM); THIi is the fixed effect of the i THI category; LNj is the fixed effect of lactation number class j; DIMQ is the quadratic fixed effect of the number of days in milk; LNj × DIMQ is the fixed effect of lactation number class j times the quadratic effect of the number of days in milk; MCk is the fixed effect of milking class k; yml is the random effect of year:month of TD; herd(cow(LN))m is the random effect of class m of lactation number nested within cow, in turn nested within farm; and eijklmn is the residual term. The random effects ym, herd(cow(LN)), and e are assumed to follow independently and identically a normal distribution of zero mean and variance σym, σherd(cow(LN)), and σe respectively. The same model was run successively for the THI from the day before the TD to 12 days before the TD. The statistical package “lme4” in the R software package (R-4.4.1) was used for this analysis [16,17].

Next, the THI solutions from the model [2] were used as dependent variables in a segmented regression to estimate a single cut-off point for the HS level on each of the 12 days prior to the TD. For this analysis, the “segmented” package in the R software was used, starting from a value of 50 < THI < 83 [18].

To determine the effect of the accumulating HS days, we calculated the number of consecutive days prior to the TD on which the cut-off points for the THI were exceeded within the window of days with significant effects for each variable of interest. If there were groups of days with non-consecutive HS, the highest value for the period was chosen. Consecutive days of HS were then categorised into 6 classes (0: 0 days of HS; 1: 1 day of HS; 2–3: 2 or 3 consecutive days of HS; 4–5: 4 or 5 consecutive days; 6–7: 6 or 7 consecutive days; and >8: 8 or more consecutive days with HS. Finally, the following mixed model was fitted:

*Yijklmn* = *CONSHSi* + *LNj* + *CONSHSi* × *LNj* + *DIMQ* + *LNj* × *DIMQ* + *MCk* + *yml* + *herd*(*cow*(*LN*))*m* + *eijklmn* (3)

where Yijklmn, LNj, DIMQ, LNj x DIMQ, Mck, k, yml, herd(cow(LN))m, and eijklmn have already been described for model [2]; CONSHSi is the fixed effect of class i of consecutive days with HS; and CONSHSi × LNj is the fixed effect of the interaction of class of consecutive days with HS with lactation number class. The least-square means (LSM) analysis of the model [3] for each class of consecutive days of HS made it possible to determine how significant the effect of accumulating consecutive days of heat stress was on the variables analysed.

## 3. Results

A mean MY of 35.69 kg/day (Table 1) was obtained between days four and 305 in milk, with a standard deviation of 9.54 kg/day. The mean value of daily milk production varied between 35.71, 35.90, 36.10, and 35.18 kg/day for the winter, spring, summer, and autumn tests, respectively. For protein, the mean results were 3.32% and 1.17 kg/day, with a standard deviation of 0.35% and 0.29 kg/day, with seasonal mean values of 3.35%, 3.31%, 3.24%, and 3.35% in winter, spring, summer, and autumn, respectively. In terms of kg of protein, the yields ranged from 1.18 kg/day in winter to 1.16 kg/day in summer. The mean milk fat in all tests was 3.90% and 1.37 kg/day, with a standard deviation of 0.93% and 0.45 kg/day up to 305 days in milk. For the winter tests, the means were 3.98% and 1.40 kg/day, with 3.89% and 1.38 kg/day for the spring, 3.77% and 1.34 kg/day in summer, and 3.94% and 1.36 kg/day in the autumn tests.

This resulted in a mean annual ECM value of 34.96 kg/day and a standard deviation of 9.20 kg/day, with a seasonal oscillation of 35.39, 35.09, 34.58, and 34.69 kg/day in winter, spring, summer, and autumn, respectively. Finally, the somatic cell count, expressed as LS, showed an overall mean of 2.47 with a standard deviation of 1.96 points. Seasonally, the value was 2.43 in winter, 2.39 in spring, 2.51 in summer, and 2.55 in autumn.

**Figure 1:**
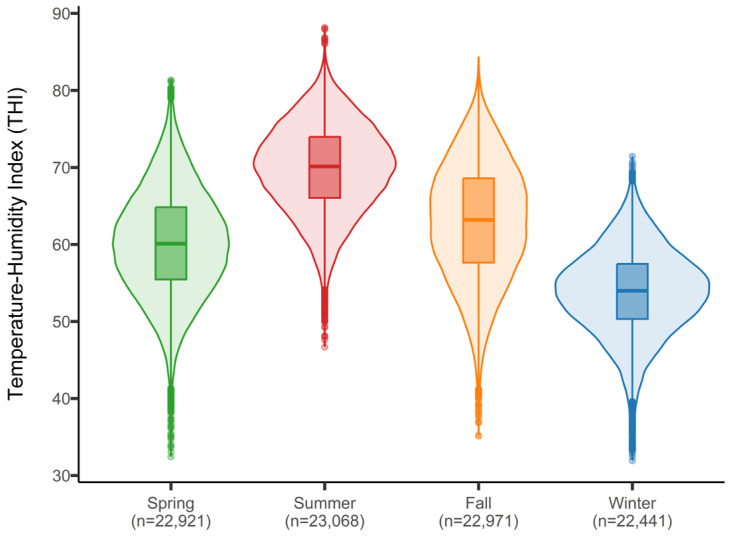
Distribution of the THI index by season between 2016 and 2021 for 46 weather stations in Galicia.

The climate values obtained from the 46 weather stations over the 6 years of the study, coinciding with the test days (TD), indicate an average daily temperature of 12.5 °C and an average humidity of 83%. The average daily maximum temperature ranged from 10.8 °C in January to 25.1 °C in August, while the average daily minimum humidity fluctuated between 53% in August and 72% in December. Based on these values, the temperature-humidity index (THI) was an average of 61 points, with a maximum of 88 and a minimum of 31 over the year. The highest values were recorded in August (71) and the lowest in January (52). According to the distribution obtained (Figure 1, Table 2), 75% of the values were below a THI of 68, 50% of the records were in the 55–68 range, and only 0.8% of the values exceeded a THI of 79.

### 3.1. Estimation of Heat Stress Effect

**Figure 2:**
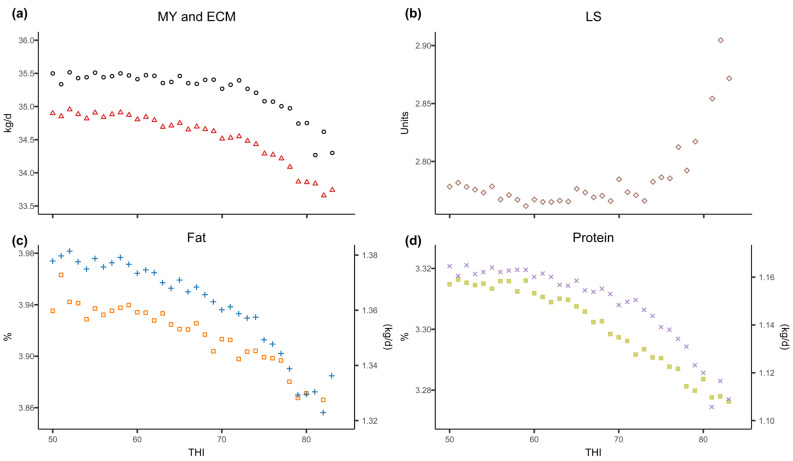
Mean fixed effects of the different temperature-humidity index (THI) categories on: (**a**) milk yield (o) and energy corrected milk (ECM) (△); (**b**) linear score (◇); (**c**) production (+) and fat percentage (□); and (**d**) yield (×) and protein percentage (🅇).

**Figure 3:**
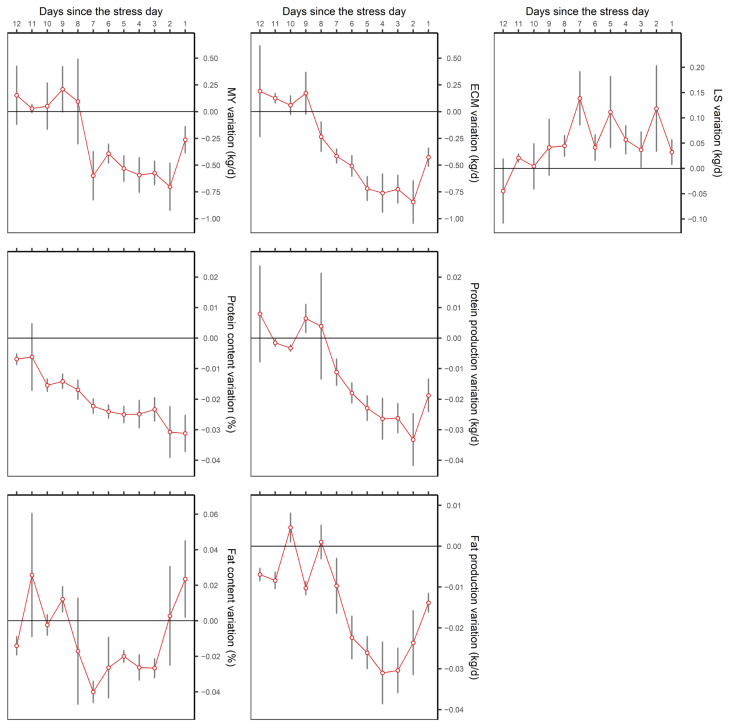
Mean effect of heat stress after exceeding the correspondent daily THI threshold for each variable with respect to the thermo-neutral zone.

The effect of heat stress (HS) was different depending on the variable studied (Figure 2) and the time at which the stressful event occurred in relation to the test day (TD) (Table 3). In relation to MY, for 7 days after the stressful event, a significant HS effect was detected (*p* < 0.0001), an effect that started from a cut-off point in the temperature-humidity index (THI) between 67 and 78 (weighted value of 72), with mean drops in milk production ranging from −0.056 kg/THI/d on the first day to −0.249 kg/THI/d on the seventh day. In this analysis, with the exception of the fourth day prior to the TD, the existence of a thermo-neutral zone was detected, with production independent of THI up to the cut-off point. From this analysis, and according to the LSM, it can be seen that when the THI > 72 threshold was exceeded, the average production losses in the thermo-neutral zone (50 < THI < 72) were highest on the fourth day after heat stress, with a drop of −0.73 kg/d (Figure 3).

The effect of the THI on milk protein percentage was apparent up to 12 days after the stress (*p* < 0.001), with a wide range of THI cut-off values causing stress, between 57 and 79 points, with a weighted value of 64. The reduction in milk protein percentage from the detected cut-off points was estimated as being between −0.001 and −0.010%/THI, although it is true that in this case downward trends are observed even from the lower THI threshold studied (50). Similar behaviour was observed in the daily protein production in kg/d, with stress influence being evident up to 7 days later (*p* < 0.001) and with a weighted cut-off point of 71, with drops in production of up to −0.008 kg/THI/d. Protein production losses estimated by LSM, when exceeding the weighted THI value (71) with respect to the mean in the interval 50 < THI < 71, reach their maximum on the third day after the stress, with a mean loss of −0.041 kg/d.

The effect of HS on milk fat percentage was mainly manifested between the third (*p* < 0.0001) and seventh (*p* < 0.0001) day, from THI values of 58 and 77 on the fifth and sixth day, respectively (weighted value THI = 63). The drop in milk fat percentage was estimated at −0.002%/THI between the third and fifth day after HS, with a maximum of −0.011%/THI on the sixth day. Fat production was affected from a weighted THI threshold of 63, with effects seen up to 7 days after the HS event. The greatest effect occurred on the fourth day after the stress, with a decrease in production of −0.006 kg/THI/d. On average, according to the LSM, when the THI was above 69, the drop in fat production relative to the average in the interval 50 < THI < 69 was again greatest on the fourth day after the HS, with a loss of −0.031 kg/d of fat.

This means that, for the variable ECM, there is a significant HS effect for the 8 days (*p* < 0.0001) after the event. This effect starts from a cut-off point in the THI between 59 and 77, with a weighted value of 68, and with mean drops in production of between −0.028 kg/THI/d on the first day and −0.161 kg/THI/d on the second day. In this case, a downward trend is also observed even from the minimum THI value studied (50), especially from the fourth day after heat stress.

The LS shows the opposite behaviour, with somatic cell counts increasing from a THI of between 73 and 81 in the first 9 days after heat stress, with a weighted value of 78. The increase in LS is estimated to be between 0.008 and 0.136 points on the sixth and second day, respectively, with HS having no detectable influence on LS in the thermo-neutral zone.

### 3.2. Accumulation of Stressful Days

Using model [3], the LSM of the number of consecutive days with HS were obtained for each productive variable (Table 4). There is a downward trend in all the variables studied except for LS. This decrease is significant (*p* < 0.05) especially after 2–3 consecutive days of HS. As the days of stress accumulate, the effects continue and are increasingly significant (*p* < 0.05), with drops of −0.9 kg/day in MY for the 6- to 7-day stress class and −0.04 kg/d of fat and protein for the same accumulated number of days with HS.

The number of consecutive days of heat stress (HS) does not impact all animals in the same manner, as shown by the significance level of the interaction between the number of consecutive days under stress and LN in model [3]. According to the lactation phase of the animal, an increase in the number of consecutive days under HS tends to decrease the values of all the variables, except for LS, where the value remains almost constant (Table 5). Therefore, stress significantly (*p* < 0.05) affects MY from 4 to 5 consecutive days of stress for the majority of the LN groups, with a reduction of approximately −0.30 to −0.40 kg/day. Contrary to what was expected and reported by model [1], the presence of a single day of stress was not significant (*p* > 0.05) in any of the lactation groups.

The percentage of protein in milk also tends to decrease in all lactation groups as consecutive days of HS increase. In this case, the protein concentration was significantly lower in the second and third lactation with even just one day of stress (*p* < 0.05). Milk protein production in kg/day was affected by the accumulation of HS days, especially in the group of cows between the second and eighth calving (*p* < 0.05), with a decrease of up to −0.02 kg/day for the group with 4–5 consecutive days of stress. As days of stress accumulate, the effect is even greater, with significant drops of up to −0.05 kg/day (*p* < 0.05) in the group of third lactation cows suffering 6–7 consecutive days of HS. Milk fat percentage shows a similar trend, with even a single day of stress affecting fat content (*p* < 0.05) in cows between their third and fifth calving, with drops of approximately −0.03%.

ECM behaves similarly to MY, with a significant influence (*p* < 0.05) seen from 4 to 5 days in almost all lactation groups, with production decreases of between −0.20 and −0.52 kg/day. The LS shows no relationship with accumulated HS, presenting very similar values even among animals with different lactation numbers.

## 4. Discussion

The climate data recorded at the weather stations reflect the temperate oceanic climate of the region (Köppen-Geiger climate classification: Cfa, Csb) [19]. The cattle in Galicia can be considered to have been exposed to mild to moderate levels of heat stress, according to the Amstrong (1994) classification for the USA [20]. However, being exposed to only mild or moderate stress conditions does not eliminate their negative effects. For example, Moore et al. (2024) found significant economic losses (−1.46$/100 L) under the effects of moderate heat stress in the Veneto region of northern Italy, despite no extreme temperature and humidity index (THI) values [21].

The average milk production, fat, and protein percentages were higher than that reported in Spanish farms with Holstein cattle (31.5 kg/day, 3.23% and 3.50%, respectively) between 1999 and 2010, according to Carabaño et al. (2016) [22]. These values also slightly exceed those found by Campos et al. (2022) [23] in two Holstein populations in Ontario and Quebec between 2010 and 2019.

The udder health status, as reflected in the LS, indicates an optimal condition, with 75% of the records below 152 cells/mL × 1000, a level considered adequate for a healthy mammary gland [24]. Furthermore, the LS follows the trend described in the literature, with somatic cell counts increasing with the age of the animal and advancing lactation period, as well as during warm months [25].

### 4.1. Effect of HS on MY, Protein, Fat, ECM, and LS

In the case of MY and its relationship with HS, two situations were defined here, a thermo-neutral zone in which the animal maintains a constant production independent of the THI, and another above the THI threshold where production declines in a linear fashion [26]. Although most authors assume linearity, some works describe a non-linear relationship between milk production and HS [22,27]. In this study, in terms of milk composition, a linear decrease in both fat and protein content was observed as the THI increased, even in the thermo-neutral zone in the case of protein, and these losses are accentuated from the critical threshold onwards. Similar behaviour, with a continuous decrease in milk fat and protein content with increasing THI, was described by Brügeman et al. (2012), Gorniak et al. (2014), and Hammami et al. (2013), although the latter did not detect the existence of a subsequent cut-off point [26,28,29].

The THI threshold determined in this study was 72 for MY, 64 and 71 for protein percentage and kg/day, and 63 and 69 for fat percentage and kg/day, while for ECM and the LS the values were 68 and 78, respectively. The value for MY coincides with that traditionally accepted in the literature as a benchmark for the Holstein breed [12,20,30], although this threshold varies considerably between studies. In Italy, Bernabucci et al. (2014) established slightly higher thresholds for MY, between 73 and 76 for Holstein cows in their first, second or third lactation, and 65–71, 72–73, and 71–72 for protein percentage, kg protein, and kg fat, respectively, without detecting a cut-off point for fat percentage [14]. On the other hand, Carabaño et al. (2016) set a THI threshold of between 74 and 83 for MY in four Holstein populations in Spain, Slovenia, Belgium, and Luxembourg, 53–64 and 62–69 for kg fat and protein, respectively, and 52–59 and 56–60 for fat and protein percentages [31]. In that case, the highest thresholds were for the Spanish region. In Canada, Campos et al. (2022) established lower cut-off points of 68 for MY, and 60 and 57 for protein and fat yield, respectively [23].

There is consensus that HS affects protein content more than fat content [32], in line with the results of this study. This is particularly evident in the more prolonged decrease in protein percentage and in the presence of a thermo-neutral zone prior to the THI limit for fat percentage. The mechanisms responsible for reducing protein production in milk due to HS are not fully understood. It is theorised that, under HS, amino acid depletion due to oxidative stress, the immune response and gluconeogenesis reduce the availability of amino acids for protein synthesis in milk [33]. The decrease in milk fat content is more associated with reduced fibre intake and changes in carbohydrate metabolism [34].

As for the somatic cell count (SCS), as in the study by Hammami et al. (2013), the THI threshold was higher than that of the other variables [26]. However, Bertocchi et al. (2014) in the Lombardy region (Italy) described the effects of HS on SCS from a THI of 57–60, substantially lower than that reported in this study [24]. A similar result was reported by Lambertz et al. (2014), where the SCS count in the THI < 65 data category was lower than in the category with THI > 65 [35].

On the other hand, they observed a prolonged HS effect on production variables over time, with repercussions lasting up to 7 days for MY, kg/day of protein, and percentage and kg/day of fat, and up to 12 days in the case of percentage protein. In Italy, Bernabucci et al. (2014) also reported that HS had an 8–12 day effect on these same variables, with a peak influence at around 3–4 days prior [14]. Similarly, Hagiya et al. (2019), in Japan, reported that the third day after stress presented the most effect in terms of milk production [36], although this was less than the 20 days reported by Souza et al. (2022) for the same variable [37]. Again, milk composition variables showed a higher HS sensitivity than MY and there was a longer-lasting effect [15,21].

In terms of milk yield and composition losses, once the THI thresholds were exceeded, drops of between −0.056 and −0.249 kg/THI/day for MY, between −0.002 and −0.008 kg/THI/day for protein, and between −0.001 and −0.006 kg/THI/day for fat were estimated. These losses for each unit increase in the THI are similar to those found by Lee et al. (2023) in multiparous Holstein cows in Korea, with decreases in MY of −0.185 kg/THI/day and −0.0085 kg/THI/day for both fat and protein production [38]. These values are also consistent with the drops in milk production reported by Brügemann et al. (2012) in Holstein cows in Germany, estimated as being between −0.16 and −0.47 kg/THI/day for different production systems, with the lower end corresponding to a confinement rearing system [28]. In the Brown Swiss breed, Maggiolino et al. (2020) quantified fat and protein losses of −0.032 and −0.053 kg/THI/day, values slightly higher than those observed in this study15. Based on our results, and in line with those described by Bernabucci et al. (2014), the third or fourth day after stress is the most critical for MY as well as for fat and protein production [14].

It is important to note that the results presented here only include data for August 2020. Since the highest THI values were recorded in that month, the actual yield losses are likely to be even higher, as the HS effect could not be estimated for a THI value of greater than 83.

### 4.2. Duration of HS Event

The intensity of the heat stress (HS) not only influences the productive performance of the animals, but it can also interact with the duration of the stress events [15,39], or even with the existence of mild nights that diminish its impact [40]. In this study we found a general trend of decreasing milk yield and milk quality as HS days accumulated. However, the accumulation of HS days showed no significant influence on LS.

Despite the unfavourable trend, the category of a single consecutive day of HS was not significant for most variables across the different lactations. This could have several explanations: on the one hand, the method used to count the days of stress, treating them in the same way regardless of the length of time from the test day; on the other hand, it is likely that the highest THI values do not occur in isolation, but are framed by hot spells lasting several days. For this reason, future studies should include an index that takes into account both HS duration and intensity [41], the distance in time from the test day (TD), and even the overnight THI [24].

It is also demonstrated that animals in more productive stages suffer the most from accumulated HS days, with 2–3 consecutive days being sufficient to significantly affect protein and fat, and 4–5 days in the case of MY. These results are similar to those obtained by Ouellet et al. (2019) in Holstein cows in the province of Quebec (Canada), who, although they found no relationship between the accumulation of consecutive days of stress and milk production, did observe effects on the ECM, protein (% and kg/d), and fat (% and kg/d) variables, with up to 6% less fat after 7–8 consecutive days of stress [42]. Heinicke et al. (2019) reported that with a persistent accumulation of 3 days of HS, cows show lower activity levels than those subjected to a single day of HS [43].

Prolonged exposure to HS can lead to metabolic imbalances and negative energy balances in cows [44,45], which may affect not only current milk production but also future reproductive performance. Several studies have highlighted reduced pregnancy rates and increased susceptibility to reproductive disorders in dairy cows exposed to HS [46,47].

### 4.3. Effect of Lactation Number

Lovarelli et al. (2024), Bernabucci et al. (2014), and Bertocchi et al. (2014) also found that cows in their first lactation are less sensitive to stress compared to cows in their second, third, or subsequent lactation [14,24,48]. This increased sensitivity of animals in more productive stages has also been observed at the individual level, with animals with a higher productive capacity being more susceptible to stress [22,49]. In terms of udder health, the animal’s lactation number tends to increase the somatic cell count in milk, although it does not seem to interact directly with the accumulation of HS days.

Furthermore, the stage of lactation determines the susceptibility of the animal to HS, with animals in higher productivity stages being more susceptible than those in early or late lactation [50]. A cow’s inability to maintain homeothermy under heat stress is mainly due to the increase in metabolic heat production associated with milk production and with the poor ability of cows to dissipate heat under hot conditions [14].

## 5. Conclusions

The north-western region of the Iberian Peninsula is not exempt from the effects of heat stress on milk production in Holstein cows, as both milk quantity and composition were affected during the period analysed. The results may be of interest to many other temperate regions that are also increasingly exposed to the effects of heat stress. Milk components such as fat and protein were more sensitive to heat than milk yield or somatic cell count. Furthermore, it is shown that the influence of heat stress on the production parameters analysed persists for several days, especially after 3–4 days. In this regard, it is also concluded that the accumulation of consecutive days of stress has an increasing impact on production, particularly in animals in higher productivity stages.

Therefore, to tackle the effects of climate change, it is necessary to design heat stress mitigation strategies for livestock, even in regions that have traditionally been less severely affected. Quantification of heat stress needs unbiased indicators (milk yield losses and milk composition or quality alteration) to estimate its economic impact and to promote preventive actions. Genetic improvements associated with stress tolerance and the adaptation of livestock facilities and animal management practices will play a key role in this context.

## Figures and Tables

**Table 1:** Daily statistics for the productive values analysed in dairy cattle in Galicia between 2016 and 2021.

	Min	Q1	Mean	Median	Q3	Max
MY (kg/d)	4.00	29.20	35.69	35.00	41.90	90.80
ECM (kg/d)	2.97	28.73	34.96	34.21	40.44	103.58
Protein (%)	2.00	3.80	3.32	3.30	3.54	5.00
Protein (kg/d)	0.11	0.98	1.17	1.16	1.36	3.19
Fat (%)	1.50	3.32	3.90	3.85	4.43	7.00
Fat (kg/d)	0.08	1.07	1.37	1.31	1.60	5.38
LS (units)	0.10	0.90	2.47	2.10	3.60	9.60

**Table 2:** Temperature, humidity, and THI index values between 2016 and 2021 at 46 meteorological stations in Galicia.

	Mean	No Data	Min	Max
Mean Temp. (°C)	12.5	5.24	−3.5	30.0
Max Temp. (°C)	17.6	6.52	−1.9	41.7
Mean Humidity (%)	83	11.3	13	100
Min Humidity (%)	62	18.2	2	100
THI	61	8.7	32	88

**Table 3:** Estimated weighted value of the maximum temperature-humidity index (THI) derived from segmented regression up to 12 days prior to the test day for milk yield and composition (protein and fat).

Days Prior		1	2	3	4	5	6	7	8	9	10	11	12	Weighted THI
Milk yield (kg/d)	BP	73	73	70	73	67	70	78						72
	a				−0.016 *									
	b	−0.056 **	−0.156 ****	−0.097 ****	−0.125 ***	−0.068 ****	−0.063 ****	−0.249 ****						
	R^2^	0.277	0.648	0.878	0.802	0.635	0.668	0.759						
Energy corrected milk (kg/d)	BP	59	72	68	73	60	70	59	77					68
	a				−0.023 ****		−0.017 ***		−0.011 ***					
	b	−0.028 ****	−0.161 ****	−0.107 ****	−0.157 ****	−0.049 ****	−0.088 ****	−0.031 ****	−0.094 ***					
	R^2^	0.416	0.773	0.906	0.901	0.817	0.919	0.648	0.669					
Protein (%)	BP	69	75	65	67	61	57	60	57	57	60	79	64	64
	a	−0.001 ****	−0.002 ****	−0.001 **	−0.001 *								−0.000 *	
	b	−0.005 ****	−0.008 ****	−0.003 ***	−0.003 ***	−0.002 ****	−0.002 ****	−0.002 ****	−0.001 ****	−0.001 ****	−0.001 ****	−0.010 **	−0.001 **	
	R^2^	0.941	0.928	0.937	0.879	0.915	0.907	0.909	0.463	0.416	0.815	0.441	0.337	
Protein (kg/d)	BP	73	74	70	73	63	63	77						71
	a	−0.001 *	−0.001 **	−0.001 **	−0.001 ***			−0.001 ****						
	b	−0.004 ***	−0.008 ****	−0.004 ****	−0.006 ****	−0.002 ****	−0.002 ****	−0.005 ***						
	R^2^	0.768	0.875	0.937	0.882	0.736	0.716	0.780						
Fat (%)	BP			61	61	58	77	58					64	63
	a						−0.002 ****							
	b			−0.002 ****	−0.002 ****	−0.002 ****	−0.011 *	−0.004 ****					−0.002 **	
	R^2^			0.477	0,341	0.606	0.740	0.819					0.226	
Fat (kg/d)	BP	58	73	68	72	60	72	76		54		59	57	69
	a		−0.001 *		−0.001 **		−0.001 ****	−0.001 ****						
	b	−0.001 ****	−0.006 ***	−0.004 ****	−0.006 ****	−0.002 ****	−0.005 ****	−0.006 *		−0.001 ****		−0.001 ***	−0.001 ***	
	R^2^	0.724	0.748	0.861	0.876	0.793	0.923	0.792		0.632		0.390	0.337	
LS (units)	BP	81	80	78	76	80	73	78	73	81				78
	a													
	b	0.033 *	0.136 ****	0.017 ***	0.016 ****	0.092 ****	0.008 **	0.072 ****	0.010 ****	0.058 **				
	R^2^	0.082	0.650	0.238	0.415	0.795	0.173	0.836	0.360	0.193				

* *p* < 0.05; ** *p* < 0.01; *** *p* < 0.001; **** *p* < 0.0001. BP = cut-off point; a = slope prior to the cut-off point; b = slope after the cut-off point.

**Table 4:** Number of database records for each category of consecutive days of stress accumulation.

	Number of Consecutive Days with Heat Stress					
	0	1	2–3	4–5	6–7	8–12
Milk yield (kg/d) THI > 72	336,846 (74%)	34,585 (8%)	44,551 (10%)	25,587 (6%)	15,596 (3%)	
Energy corrected milk (kg/d) THI > 68	261,428 (57%)	33,193 (7%)	54,086 (12%)	42,094 (9%)	38,142 (8%)	28,222 (6%)
Protein (%) THI > 64	189,584 (41%)	29,841 (7%)	42,552 (9%)	39,581 (9%)	34,693 (8%)	120,914 (18%)
Protein (kg/d) THI > 71	319,282 (70%)	34,690 (8%)	49,722 (11%)	30,794 (7%)	22,677 (5%)	
Fat (%) THI > 64	217,635 (48%)	39,175 (9%)	70,538 (15%)	129,817 (28%)		
Fat (kg/d) THI > 68	268,576 (59%)	34,238 (7%)	56,371 (12%)	44,887 (10%)	53,093 (12%)	
Linear Score (units) THI > 78	436,747 (96%)	11,438 (3%)	7379 (2%)	1555 (0%)	46 (0%)	

**Table 5:** Least-squared means for milk production and composition (fat and protein), energy corrected milk (ECM), and linear score (LS) versus lactation number and number of consecutive days with heat stress in the period 2016–2021 in Galicia.

	CONSEC HS	Milk Yield (kg/d)	ECM (kg/d)	Protein (%)	Protein (kg/d)	Fat (%)	Fat (kg/d)	LS (units)
LN = 1	0	33.67 ^a^	32.15 ^a^	3.30 ^a^	1.11 ^a^	3.67 ^a^	1.22 ^a^	1.89 ^a^
	1	33.52 ^ab^	32.11 ^ab^	3.30 ^a^	1.11 ^a^	3.67 ^a^	1.22 ^a^	1.93 ^ab^
	2–3	33.56 ^ab^	32.00 ^ab^	3.30 ^a^	1.10 ^a^	3.68 ^a^	1.22 ^a^	1.99 ^b^
	4–5	33.41 ^b^	31.95 ^b^	3.30 ^a^	1.09 ^b^	3.64 ^b^	1.21 ^a^	1.99 ^ab^
	6–7	33.08 ^c^	31.66 ^c^	3.29 ^a^	1.09 ^b^		1.20 ^b^	2.16 ^ab^
	8+		31.61 ^c^	3.28 ^b^				
LN = 2	0	39.10 ^a^	37.40 ^a^	3.30 ^a^	1.28 ^a^	3.71 ^a^	1.43 ^a^	2.24 ^a^
	1	39.23 ^a^	37.36 ^ab^	3.29 ^b^	1.27 ^b^	3.68 ^ab^	1.43 ^a^	2.27 ^a^
	2–3	39.03 ^ab^	37.36 ^a^	3.29 ^b^	1.27 ^b^	3.68 ^b^	1.43 ^a^	2.30 ^a^
	4–5	38.86 ^b^	37.13 ^b^	3.28 ^bc^	1.25 ^c^	3.67 ^c^	1.41 ^b^	2.21 ^a^
	6–7	38.24 ^c^	36.69 ^c^	3.28 ^c^	1.25 ^c^		1.39 ^c^	2.10 ^a^
	8+		36.57 ^c^	3.25 ^d^				
LN = 3	0	40.64 ^a^	38.79 ^a^	3.27 ^a^	1.32 ^a^	3.70 ^a^	1.49 ^a^	2.74 ^a^
	1	40.72 ^a^	38.82 ^a^	3.26 ^b^	1.31 ^a^	3.67 ^b^	1.50 ^a^	2.76 ^a^
	2–3	40.49 ^ab^	38.63 ^a^	3.26 ^b^	1.30 ^b^	3.67 ^b^	1.48 ^b^	2.78 ^a^
	4–5	40.26 ^b^	38.34 ^b^	3.25 ^bc^	1.29 ^c^	3.66 ^b^	1.46 ^c^	2.71 ^a^
	6–7	39.70 ^c^	38.01 ^c^	3.24 ^c^	1.27 ^d^		1.44 ^d^	2.09 ^a^
	8+		37.71 ^d^	3.22 ^d^				
LN = 4–5	0	40.34 ^a^	38.45 ^a^	3.25 ^a^	1.30 ^a^	3.70 ^a^	1.48 ^a^	3.21 ^a^
	1	40.31 ^ab^	38.40 ^a^	3.25 ^a^	1.29 ^b^	3.67 ^bc^	1.47 ^ab^	3.19 ^a^
	2–3	40.05 ^bc^	38.04 ^b^	3.23 ^b^	1.28 ^b^	3.68 ^b^	1.46 ^b^	3.33 ^b^
	4–5	39.84 ^c^	37.95 ^b^	3.23 ^bc^	1.26 ^c^	3.65 ^c^	1.45 ^c^	3.34 ^ab^
	6–7	39.33 ^d^	37.53 ^c^	3.22 ^c^	1.26 ^c^		1.43 ^d^	3.45 ^ab^
	8+		37.25 ^c^	3.20 ^d^				
LN = 6–8	0	38.36 ^a^	36.69 ^a^	3.23 ^a^	1.23 ^a^	3.75 ^a^	1.42 ^a^	3.72 ^a^
	1	38.19 ^ab^	36.69 ^ab^	3.22 ^ab^	1.22 ^ab^	3.76 ^a^	1.40 ^abc^	3.88 ^a^
	2–3	37.99 ^ab^	36.31 ^ab^	3.22 ^ab^	1.21 ^b^	3.68 ^b^	1.40 ^ab^	3.43 ^b^
	4–5	37.50 ^b^	36.17 ^bc^	3.21 ^ab^	1.19 ^c^	3.69 ^b^	1.38 ^bc^	3.54 ^ab^
	6–7	37.36 ^b^	35.64 ^c^	3.20 ^b^	1.18 ^c^		1.37 ^c^	-
	8+		35.47 ^c^	3.18 ^c^				

CONS HS: number of consecutive days with heat stress; LN: lactation number. ^a–d^: Means within the same lactation number with different superscripts are statistically significant (*p* < 0.05).

## Data Availability

The data presented in this study are available on request from the corresponding author, due to confidentiality or privacy concerns.
